# Development of a nomogram for predicting 2-year native liver survival in biliary atresia using dynamic liver function indicators

**DOI:** 10.3389/fped.2026.1636257

**Published:** 2026-02-26

**Authors:** Bingliang Li, Hongxia Ren

**Affiliations:** 1 Department of Neonatal Surgery, Shanxi Medical University Affiliated Children’s Hospital, Taiyuan, China; 2 Department of Pediatrics, Shanxi Medical University, Taiyuan, China

**Keywords:** biliary atresia, dynamic liver function indicators, native liver survival, nomogram, prognostic prediction model

## Abstract

**Objectives:**

To develop and validate a nomogram based on dynamic liver function indexes for predicting native liver survival (NLS) in children with biliary atresia (BA) at 2 years post-Kasai surgery, providing clinicians with a basis for individualized treatment decisions and optimizing early intervention strategies for high-risk children.

**Methods:**

Children with type III BA were categorized by their 2-year NLS status. Univariate and multivariate logistic regression analyses were performed to identify predictors of NLS and to construct a nomogram model. Model performance was evaluated using internal bootstrap validation (1,000 resamples) and a training–test split (7:3), with discrimination assessed by the area under the receiver operating characteristic curve (AUC) and calibration by calibration curves.

**Results:**

A total of 134 children with type III BA were included. Univariate analysis identified significant associations between prognosis and the following: age at surgery, jaundice clearance failure, liver fibrosis stage, and 3-month postoperative levels of gamma-glutamyl transpeptidase (GGT), serum albumin (ALB), and aspartate aminotransferase and platelet ratio index (APRI) (all *P* < 0.05). Multivariate analysis established these independent predictors: liver fibrosis stage F4 (OR = 3.418, 95% CI: 1.745–6.695), APRI at 3 months (OR = 2.285, 95% CI: 1.175–4.445), age at surgery (OR = 1.773, 95% CI: 1.192–2.637), GGT at 3 months (OR = 1.942, 95% CI: 1.211–3.117), ALB at 3 months (OR = 0.948, 95% CI: 0.916–0.981), and jaundice clearance failure (OR = 2.437, 95% CI: 1.275–4.657). The resulting nomogram demonstrated stable performance across age subgroups (AUC = 0.926 for ≤60 days; AUC = 0.867 for >60 days). In the training set, the AUC was 0.872 (95% CI: 0.813–0.931), with sensitivity of 90.7% and specificity of 78.8%. The model showed excellent generalizability in the independent test set (AUC = 0.971).

**Conclusions:**

This study developed and validated a nomogram integrating dynamic liver function indicators, effectively predicting 2-year NLS in children with BA. The model provides a reliable quantitative basis for individualized treatment decisions and early intervention, with strong clinical applicability, particularly in resource-limited settings. It offers a foundation for optimizing early intervention strategies for high-risk children.

## 1 Introduction

Biliary atresia (BA) is a severe hepatobiliary disorder of infancy characterized by progressive fibroinflammatory obstruction of the extrahepatic and intrahepatic bile ducts (1). If left untreated, most affected infants develop cirrhosis before the age of two years (2). Kasai portoenterostomy (KPE) remains the first-line treatment for BA; however, progressive liver fibrosis may still occur after surgery, leading to reduced native liver survival (NLS) and poor long-term outcomes. Approximately 40%–60% of children eventually require liver transplantation within 2 years after KPE (3). Therefore, accurate prediction of 2-year NLS after KPE is clinically important for early treatment planning and improved survival outcomes.

Currently, prognostic assessment of BA primarily relies on static indicators, including age at surgery, liver fibrosis stage, and postoperative jaundice clearance (JC) (4–10). However, the predictive value of these single-point indicators is limited, as they fail to capture postoperative dynamic changes in liver function or reflect the balance between fibrosis progression and hepatic functional compensation.

In recent years, several studies have attempted to evaluate long-term outcomes in children with BA using postoperative biochemical markers or imaging techniques such as liver elastography (11). Nevertheless, existing predictive models still have notable limitations. Most models incorporate only static variables or a single dynamic indicator and lack comprehensive analysis of liver function changes at key postoperative time points. In addition, some models rely on costly equipment or specialized biomarker testing, limiting their applicability in primary healthcare settings. Moreover, many previous studies are based on traditional regression models with limited visualization and individualized prediction capabilities. Nomograms, as multivariable predictive tools, allow graphical visualization of the contribution of multiple factors to clinical outcomes and have been widely applied in various fields. However, their application in prognostic prediction for BA remains limited. In this study, dynamic liver function indicators were integrated with static clinical and pathological variables to construct a nomogram for predicting 2-year NLS in children with BA, aiming to improve early risk stratification and provide a practical, non-invasive, and generalizable prognostic tool, particularly for resource-limited settings.

## 2 Materials and methods

### 2.1 Patients

This retrospective study enrolled children with type III biliary atresia (BA) who underwent Kasai portoenterostomy with intraoperative liver biopsy at Shanxi Provincial Children's Hospital between January 2013 and May 2023.The inclusion criteria were as follows: (1) confirmed diagnosis of type III BA by intraoperative exploration and/or cholangiography; (2) surgery performed by the same surgical team with intraoperative liver biopsy; (3) availability of complete clinical, laboratory, and pathological data. The exclusion criteria were: (1) perioperative death due to severe comorbidities (e.g., congenital heart disease or severe metabolic disorders); (2) absence of liver pathological examination; (3) incomplete clinical or laboratory data. This study was approved by the Ethics Committee of Shanxi Provincial Children's Hospital (IRB-WZ-2024-031). Written informed consent was obtained from the legal guardians of all participants, and the requirement for written consent was waived where applicable.

### 2.2 Observation indicators

Clinical data were collected, including: (1) demographic characteristics (sex and age at surgery, in days); (2) histopathological staging of liver fibrosis; (3) biochemical parameters measured within 3 days before surgery and at 1, 2, and 3 months postoperatively, including alanine aminotransferase (ALT), aspartate aminotransferase (AST), gamma-glutamyl transpeptidase (GGT), serum albumin (ALB), total bilirubin (TBIL), direct bilirubin (DBIL), total bile acid (TBA), alkaline phosphatase (ALP), and platelet count (PLT). The aspartate aminotransferase and platelet ratio index (APRI) was calculated as a serum aspartate aminotransferase level (U/L)/upper limit of normal × 100/platelet count (10^9^/L) (12), and the upper limit of normal for AST was set at 40 U/L.

Jaundice clearance was defined as a serum TBIL level <34.2 μmol/L at 3 months postoperatively, according to established clinical criteria and previous studies (13). Cholangitis was defined as an episode of unexplained fever (≥38°C) combined with at least one of the following: recurrent jaundice or acholic stools; DBIL ≥20 μmol/L; white blood cell count ≥10 × 10⁹/L or C-reactive protein ≥10 mg/L (14). Liver fibrosis was assessed on Masson-stained sections under light microscopy and staged according to the METAVIR scoring system (F0–F4) (15). All specimens were independently evaluated by two experienced pathologists, and any discrepancies were resolved by consultation with a third senior pathologist.

For laboratory variables with missing values at certain postoperative time points, the missing data pattern was examined and assumed to be completely at random; therefore, a complete-case analysis approach was adopted.

### 2.3 Case grouping

The primary outcome was native liver survival (NLS) at 2 years after Kasai portoenterostomy. Patients were categorized into the NLS group (survival with native liver at 2 years) and the non-NLS group (death or liver transplantation within 2 years postoperatively). Indications for liver transplantation referral followed established clinical guidelines for pediatric end-stage liver disease (16, 17).

### 2.4 Statistical analysis

Statistical analyses were performed using SPSS version 26.0 and R version 4.4.3. Normality of continuous variables was assessed using the Shapiro–Wilk test. Normally distributed variables were expressed as mean ± standard deviation and compared using Student's *t*-test, whereas non-normally distributed variables were expressed as median (interquartile range) and compared using the Mann–Whitney *U*-test. Categorical variables were presented as frequencies and percentages and compared using the chi-square test or Fisher's exact test, as appropriate.

Univariate logistic regression analysis was used to identify candidate predictors of 2-year NLS. Variables showing statistical significance were further evaluated in multivariate logistic regression analysis, with attention to clinical relevance and multicollinearity. Odds ratios (ORs) and 95% confidence intervals (CIs) were calculated.

A nomogram was constructed based on independent predictors using the R package rms. Model performance was evaluated in terms of discrimination, calibration, and clinical utility. Discrimination was assessed by calculating the area under the receiver operating characteristic curve (AUC), and calibration was evaluated using calibration curves and the Hosmer–Lemeshow goodness-of-fit test. Internal validation was performed using bootstrap resampling with 1,000 iterations. Additionally, the dataset was randomly divided into training and testing sets at a ratio of 7:3 to assess model generalizability. Decision curve analysis was conducted to evaluate clinical usefulness. Subgroup analyses were performed according to age at surgery (≤60 days and >60 days). All statistical tests were two-sided, and *P* < 0.05 was considered statistically significant.

## 3 Results

### 3.1 Baseline characteristics of the study population

A total of 134 children were included and categorized into the NLS group (*n* = 80) and the non-NLS group (*n* = 54). The distribution of liver fibrosis stages in the entire cohort was F1 (*n* = 23), F2 (*n* = 54), F3 (*n* = 24), and F4 (*n* = 33). No significant differences were observed between the two groups in terms of sex or the incidence of cholangitis (both *P* > 0.05). In contrast, age at surgery, jaundice clearance rate, and liver fibrosis stage distribution differed significantly between the two groups (all *P* < 0.05; Table 1).

**Table 1:** Baseline characteristics of infants with biliary atresia stratified by 2-year native liver survival status.

Variable	NLS Group (*n* = 80)	Non-NLS Group (*n* = 54)	*P*
Age at surgery (in days)	61.2 ± 15.6	70.7 ± 20.5	
≤60 days	42 (52.5)	20 (37.0)	0.003
＞60 days	38 (47.5)	34 (63.0)	
Sex [n (%)]			
Male	34 (42.5)	22 (40.7)	0.84
Female	46 (57.5)	32 (59.3)	
Postoperative jaundice clearance [n (%)]			
Yes	45 (56.3)	18 (33.3)	0.009
No	35 (43.7)	36 (66.7)	
Occurrence of cholangitis [n (%)]			
Yes	40 (50.0)	29 (53.7)	0.674
No	40 (50.0)	25 (46.3)	
Grading of liver fibrosis [n (%)]			
F1	22 (27.5)	1 (1.9)	<0.001
F2	38 (47.5)	16 (29.6)	
F3	10 (12.5)	14 (25.9)	
F4	10 (12.5)	23 (42.6)	

### 3.2 Dynamic trend of preoperative and postoperative laboratory indicators

Laboratory indicators were compared between the two groups at predefined time points, including preoperative baseline and postoperative months 1, 2, and 3. The results showed that the differences in GGT, ALB, and TBIL between the two groups of children were statistically significant at postoperative months 1, 2, and 3, while APRI showed significant statistical differences preoperatively and at postoperative months 1, 2, and 3. Detailed comparisons of laboratory indicators at each time point are presented in Table 2.

**Table 2:** Dynamic changes in key liver function indicators from preoperative baseline to 3 months after Kasai portoenterostomy, stratified by 2-year native liver survival.

Indexes	Time points	NLS Group (*n* = 80)	Non-NLS Group (*n* = 54)	*P*
GGT (U/L)	Preoperatively	374.0 (274.5, 728.0)	319.5 (208.5, 438.0)	0.113
	One month postoperatively	675.0 (506.5, 830.0)	1,050.0 (781.5, 1,303.0)	<0.001
	Two month postoperatively	646.5 (419.5, 860.0)	1,009.5 (682, 1,332.5)	<0.001
	Three month postoperatively	365.5 (179.5, 645.5)	946.0 (630.0, 1,408.0)	<0.001
*P*	–	<0.001	<0.001	
ALB (g/L)	Preoperatively	36.6 (33.3, 39.1)	37.1 (34.4, 38.8)	0.503
	One month postoperatively	38.5 (35.0, 41.9)	35.3 (31.5, 39.6)	<0.001
	Two month postoperatively	39.9 (35.2, 42.6)	36.1 (31.5, 39.9)	<0.001
	Three month postoperatively	38.8 (35.6, 41.3)	35.5 (32.2, 40.1)	0.005
*P*	–	0.003	0.363	
TBIL (*μ*mol/L)	Preoperatively	192.7 (153.1, 226.4)	181.7 (163.7, 245.2)	0.789
	One month postoperatively	110.6 (57.0, 163.5)	140.7 (97.1, 173.7)	0.024
	Two month postoperatively	44.7 (15.4, 114.7)	90.8 (30.8, 153.4)	0.010
	Three month postoperatively	29.9 (9.8, 78.2)	61.9 (20.9, 126.0)	0.007
*P*	–	<0.001	<0.001	
APRI	Preoperatively	1.035 (0.789, 1.313)	1.348 (1.106, 2.016)	<0.001
	One month postoperatively	0.603 (0.420, 0.944)	0.908 (0.614, 1.250)	0.002
	Two month postoperatively	1.091 (0.737, 1.563)	1.464 (0.997, 2.141)	0.002
	Three month postoperatively	0.896 (0.689, 1.359)	1.368 (1.037, 2.007)	<0.001
*P*	–	<0.001	<0.001	

### 3.3 Univariate logistic regression analysis to screen predictive factors

To identify potential predictors of 2-year NLS after Kasai portoenterostomy, we conducted univariate logistic regression analysis on all candidate variables. The results showed that age at surgery, jaundice clearance failure, liver fibrosis F4 stage, GGT at 2 and 3 months postoperatively, ALB at 2 and 3 months postoperatively, TBIL at 3 months postoperatively, and APRI at 3 months postoperatively were significantly associated with outcomes (all *P* < 0.05). In contrast, factors such as gender and occurrence of cholangitis were not statistically significant (all *P* > 0.05). Detailed results are presented in Table 3. To avoid redundancy, the clinically established dichotomous variable “jaundice clearance failure” was prioritized over the continuous variable “TBIL at 3 months postoperatively” in the multivariate analysis.

**Table 3:** Univariate logistic regression analysis of factors associated with 2-year native liver survival after Kasai portoenterostomy.

Variable	OR	95% CI	*P*
Age at surgery	1.894	1.420–2.526	<0.001
Liver fibrosis stage			
F2	1.183	0.402–3.486	0.760
F3	1.326	0.550–3.196	0.529
F4	2.947	1.240–7.005	0.014
Occurrence of cholangitis	1.160	0.581–2.361	0.674
Jaundice clearance failure	2.618	1.650–4.154	<0.001
GGT at 1m	1.000	0.999–1.000	0.483
GGT at 2m	1.001	1.001–1.002	0.007
GGT at 3m	2.076	1.158–3.720	0.014
ALB at 1m	1.042	0.956–1.137	0.350
ALB at 2m	0.92	0.846–1.000	0.049
ALB at 3m	0.896	0.834–0.962	0.002
TBIL at 1m	0.999	0.993–1.006	0.770
TBIL at 2m	0.996	0.986–1.005	0.355
TBIL at 3m	1.004	1.001–1.013	0.037
APRI at preoperative	0.513	0.161–1.633	0.258
APRI at 1m	1.623	0.685–3.847	0.271
APRI at 2m	1.005	0.998–1.011	0.156
APRI at 3m	2.159	1.203–3.876	0.010

### 3.4 Multivariate logistic regression analysis

**Figure 1:**
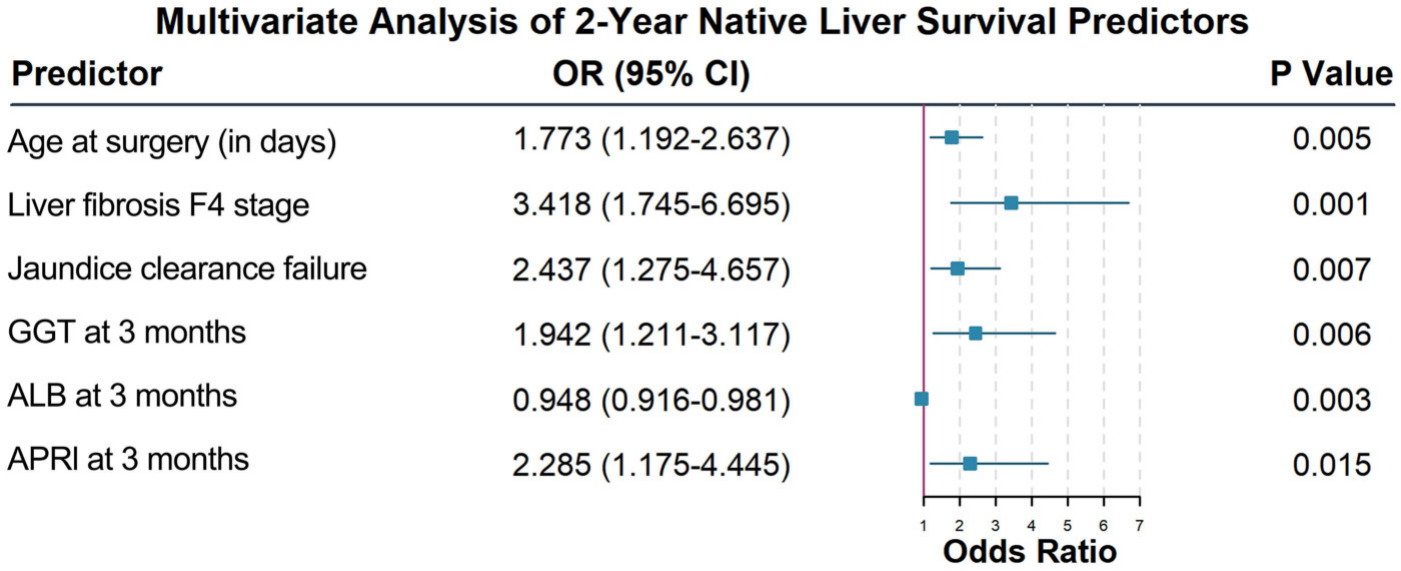
Forest plot of independent predictors of 2-year native liver survival derived from multivariate logistic regression.

Indicators with statistical significance in univariate analysis were included in the multivariate model, and the results showed that liver fibrosis stage F4 (OR = 3.418, 95% CI: 1.745–6.695), postoperative 3-month APRI (OR = 2.285, 95% CI: 1.175–4.445), age at surgery (OR = 1.773, 95% CI: 1.192–2.637), postoperative 3-month GGT (OR = 1.942, 95% CI: 1.211–3.117), postoperative 3-month ALB (OR = 0.948, 95% CI: 0.916–0.981), and failure of jaundice clearance (OR = 2.437, 95% CI: 1.275–4.657) were independent predictors of 2-year native liver survival in children with BA (all *P* < 0.05). The forest plot (Figure 1) shows the effect size of each factor.

### 3.5 Nomogram development

**Figure 2:**
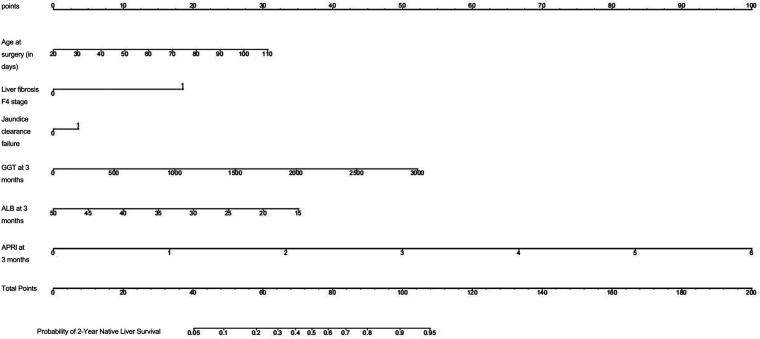
Nomogram for individualized prediction of 2-year native liver survival after Kasai portoenterostomy in infants with biliary atresia.

**Figure 3:**
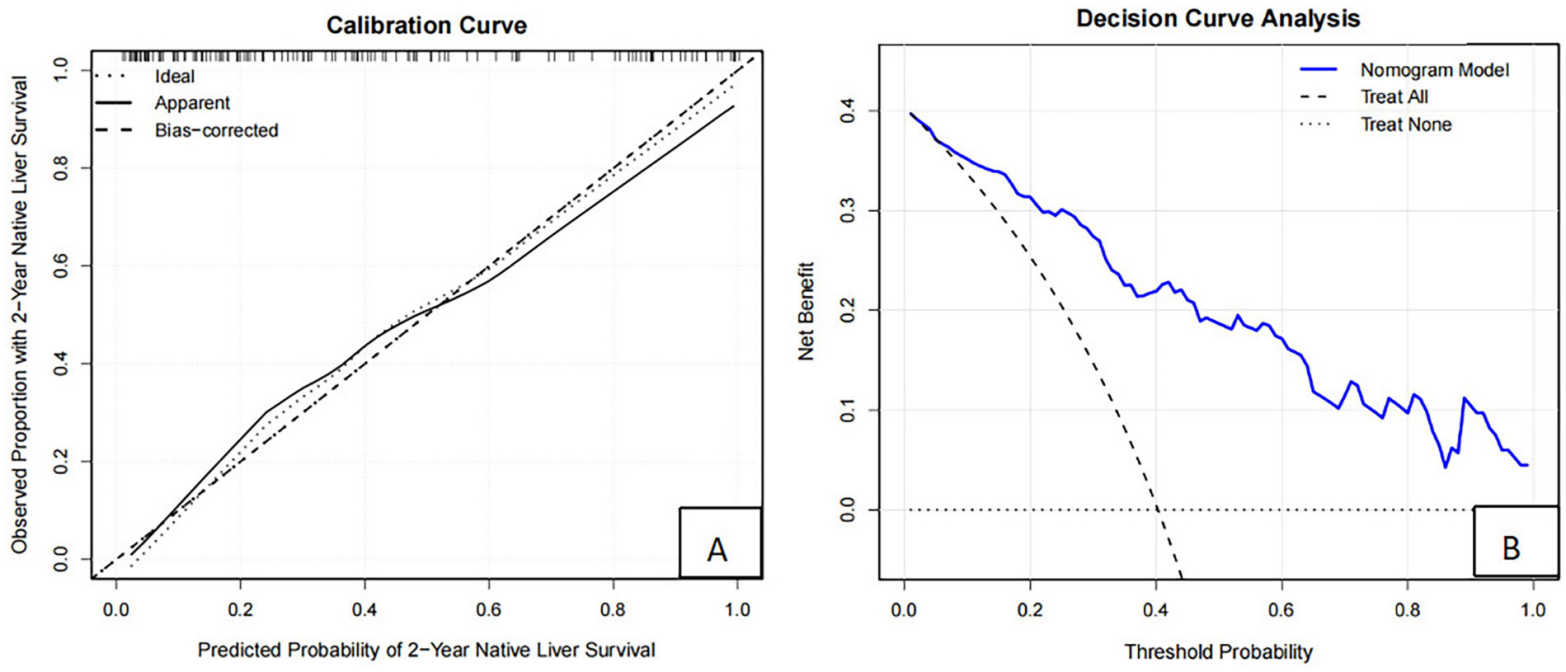
Calibration and clinical utility of the nomogram for predicting 2-year native liver survival. **(A)** Calibration curve comparing predicted and observed probabilities. **(B)** Decision curve analysis demonstrating net clinical benefit across threshold probabilities.

**Figure 4:**
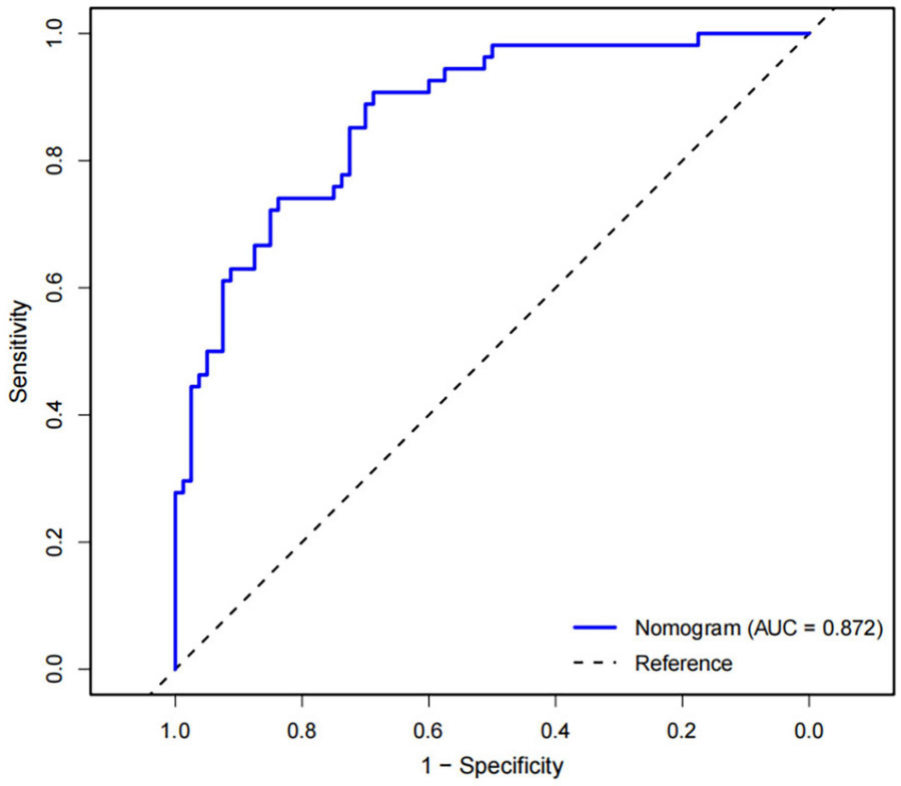
ROC curve of the nomogram for predicting 2-year native liver survival.

Based on independent predictors such as liver fibrosis stage, jaundice clearance, age at surgery, GGT, ALB, APRI at 3 months after surgery, a nomogram model for predicting 2-year NLS was constructed (Figure 2), and the calibration curve showed that the prediction curve fit well with the ideal prediction curve (Figure 3A), indicating good agreement between the predicted probabilities and the observed 2-year NLS outcomes, and the decision curve showed that the prediction of 2-year NLS nomogram had great clinical application value (Figure 3B). The 2-year NLS nomogram model had an AUC of 0.872 (95% CI: 0.813–0.931), with a sensitivity of 90.7% and specificity of 78.8%; its AUC was higher than the AUCs of other indicators when used alone for prediction (DeLong test, all *P*-values < 0.05) (Figure 4).

### 3.6 Age subgroup analysis

**Figure 5:**
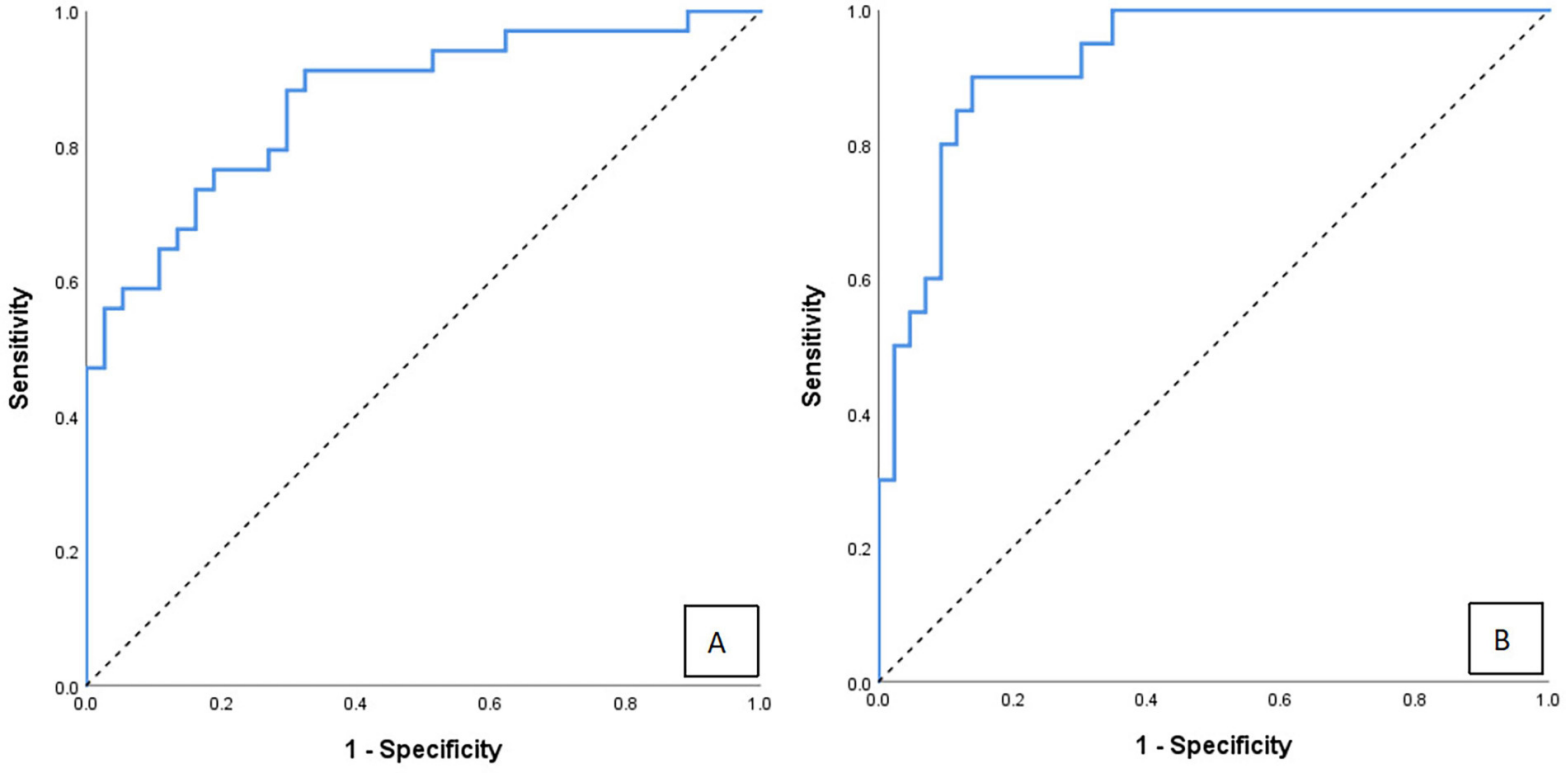
Age-stratified ROC analysis of the nomogram for predicting 2-year native liver survival. **(A)** Infants operated at >60 days of age. **(B)** Infants operated at ≤60 days of age.

To assess the predictive performance of the nomogram model in children of different age at surgery, we performed multivariate logistic regression analysis on each predictive factor using 60 days as the cutoff (Table 4). In the subgroup with age at surgery >60 days (*n* = 63), the nomogram model predicting 2-year native liver survival had an AUC of 0.867 (95% CI: 0.783–0.951), with a sensitivity and specificity of 91.2% and 67.6%, respectively (Figure 5A). The Hosmer-Lemeshow test also indicated good calibration (χ^2^ = 11.898, df = 8, *P* = 0.156). In the subgroup with age at surgery ≤60 days (*n* = 71), the model had an AUC of 0.926 (95% CI: 0.863–0.989), with a sensitivity and specificity of 90.0% and 86.0%, respectively (Figure 5B), and the Hosmer-Lemeshow test showed good calibration for this subgroup (χ^2^ = 3.817, df = 8, *P* = 0.817). Calibration curves in both subgroups demonstrated good fitting. These results indicate that the nomogram model developed in this study has good discrimination and calibration ability in children with BA across different age at surgery, with stable predictive performance.

**Table 4:** Age-stratified multivariate logistic regression analysis (≤60 vs. >60 days at surgery) of predictors of 2-year native liver survival.

Variable	Age at surgery ≤60 days		Age at surgery >60 days	
	OR (95% CI)	*P*	OR (95% CI)	*P*
Age at surgery	1.512 (1.126–2.030)	0.006	1.670 (1.136–2.455)	0.009
Liver Fibrosis F4 stage	3.339 (1.293–8.622)	0.013	4.161 (1.321–13.107)	0.015
Jaundice Clearance failure	2.568 (0.838–7.869)	0.097	2.211 (1.101–4.440)	0.026
GGT at 3m	1.802 (1.175–2.764)	0.007	1.795 (1.120–2.877)	0.015
ALB at 3m	0.845 (0.754–0.947)	0.004	0.905 (0.831–0.986)	0.021
APRI at 3m	2.313 (1.354–3.951)	0.002	2.067 (1.057–4.041)	0.034

### 3.7 Training set/test set validation results

**Figure 6:**
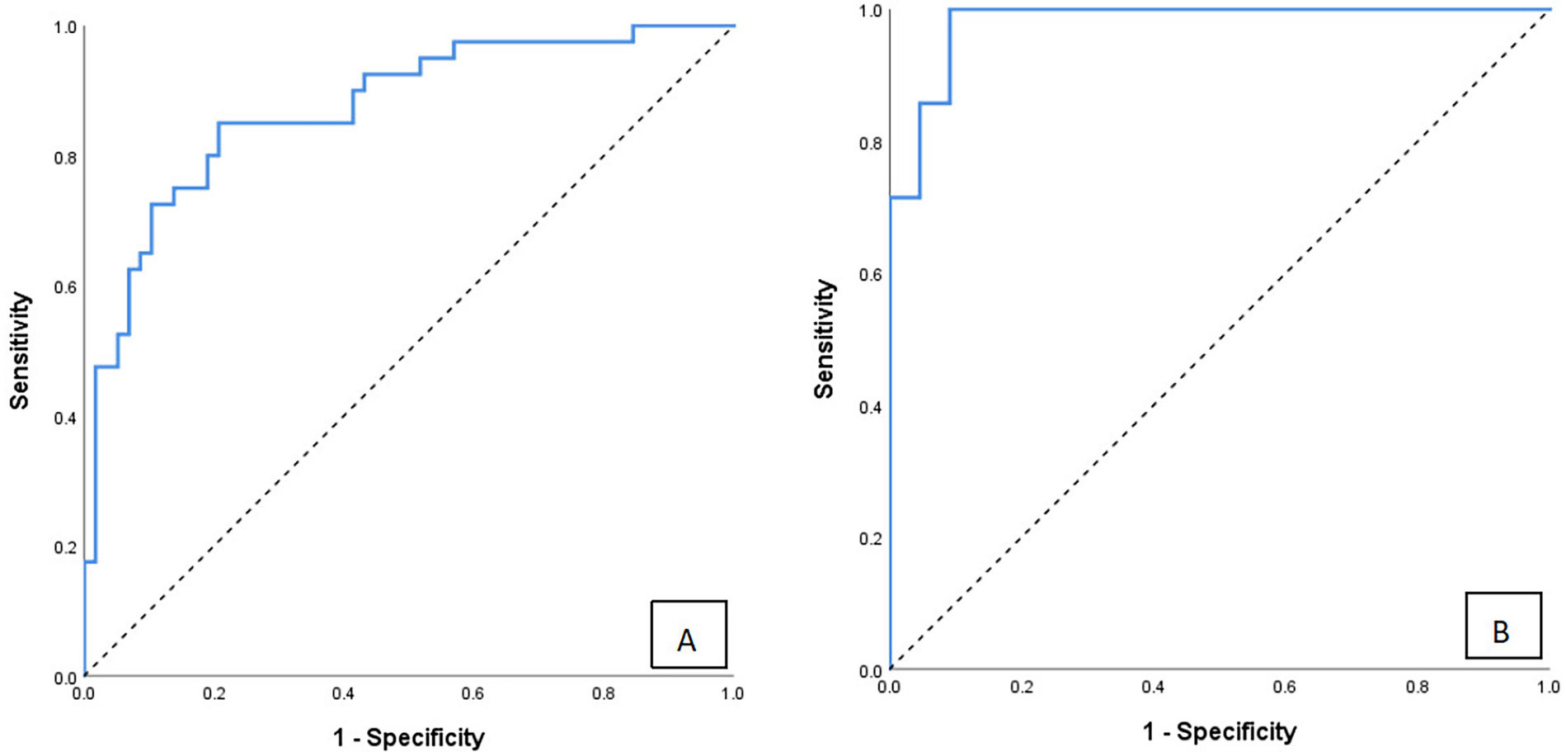
ROC curves of the nomogram for predicting 2-year native liver survival in the training and test cohorts. **(A)** Training cohort. **(B)** Test cohort.

To further objectively evaluate the generalization ability of the model, we randomly divided the total dataset into a training set (*n* = 98, 73.1%) and a test set (*n* = 36, 26.9%), and reconstructed multivariable logistic regression models in both the training and test sets (Table 5). The nomogram model reconstructed in the training set demonstrated excellent predictive performance, with an AUC of 0.872 (95% CI: 0.800–0.945), sensitivity and specificity of 85.0% and 79.3%, respectively (Figure 6A). The Hosmer-Lemeshow test for the training set indicated good calibration (χ^2^ = 7.285, df = 8, *P* = 0.506). When applied to the independent test set, the model achieved an AUC of 0.971 (95% CI: 0.946–1.000), with sensitivity and specificity of 100% and 90.9%, respectively (Figure 6B). The Hosmer-Lemeshow test for the test set also showed good calibration (χ^2^ = 4.916, df = 8, *P* = 0.670). The similar performance between the training and test sets suggests a low risk of overfitting and good external validity of the model.

**Table 5:** Multivariate logistic regression analysis of predictors of 2-year native liver survival in training and test cohorts.

Variable	Training set		Test set	
	OR (95% CI)	*P*	OR (95% CI)	*P*
Age at surgery	1.823 (1.065–3.121)	0.029	1.728 (1.109–2.694)	0.016
Liver Fibrosis F4 stage	3.367 (1.418–7.996)	0.006	3.414 (1.150–10.136)	0.027
Jaundice clearance failure	3.280 (1.627–6.613)	0.001	3.062 (0.985–9.518)	0.053
GGT at 3m	1.864 (1.315–2.933)	0.001	1.719 (1.043–2.834)	0.034
ALB at 3m	0.964 (0.916–1.015)	0.136	0.915 (0.857–0.977)	0.009
APRI at 3m	2.743 (1.318–5.710)	0.007	2.295 (1.056–4.987)	0.036

## 4 Discussion

Biliary atresia (BA) is a neonatal liver disease characterized by progressive fibrous occlusion of the biliary tract and is the most common surgical cause of neonatal cholestasis (2). Although successful KPE can achieve local biliary drainage in about 60% of children in the early postoperative period, cholestasis is difficult to improve due to persistent intrahepatic microbile duct lesions and portal artery inflammation, and progressive liver fibrosis and even cirrhosis may still occur (18), which markedly compromises long-term native liver survival. Therefore, early and accurate prediction of 2-year NLS in children with BA may facilitate individualized long-term treatment strategies (19). In this study, the clinical data of 134 children with type III BA were retrospectively analyzed, and the prognosis prediction model was constructed by integrating preoperative, intraoperative and postoperative multi-dimensional parameters. A nomogram model was developed by integrating the histological stage of liver fibrosis (assessed by intraoperative biopsy), age at surgery, jaundice clearance status at 3 months, and dynamic biochemical indicators (GGT, ALB, and APRI) measured at 3 months postoperatively. This model demonstrated good potential for clinical application in predicting 2-year native liver survival. Except for the liver fibrosis staging results, which were determined by intraoperative liver biopsy of KPE, all other parameters were obtained by non-invasive methods, which improved the clinical applicability of the model. The nomogram model showed excellent predictive power (AUC = 0.872), and its discriminant power was significantly better than that of a single clinical indicator, and internal validation using bootstrap resampling (1,000 iterations) demonstrated good agreement between the predicted and observed outcomes, and the decision curve analysis confirmed that the model had a significant clinical net benefit within a wide threshold probability range.

Although several studies (11, 20–23) have suggested that preoperative liver biopsy and liver elastography can predict the prognosis of KPE surgery by assessing the degree of liver fibrosis, the invasive procedure of liver biopsy may increase the risk of trauma in children, and the limited amount of liver tissue sampling in infants can lead to bias in pathologic evaluation (21). Although liver ultrasound elastography is a non-invasive test, most primary hospitals are not equipped with elastography in ultrasound equipment, which limits its clinical application. Notably, Chi et al. (24) proposed that dynamic monitoring of matrix metalloproteinase-7 (MMP-7) after Kasai has early prognostic value. They found that changes in serum MMP-7 concentrations at 6 weeks postoperatively were a significant predictor of native liver survival at 2 years postoperatively. However, the cost of such special biomarker testing is high and most of them rely on delivery testing platforms, so it is not feasible to implement them in areas with poor medical resources. Based on this, this study aimed to construct a nomogram prediction model based on routine clinically accessible parameters, thereby reducing reliance on specialized equipment or high-cost biomarker testing by integrating preoperative baseline characteristics (surgical age), intraoperative histopathological evidence (liver fibrosis staging), and postoperative routine follow-up indicators (JC status, GGT, ALB, APRI). This model is especially suitable for primary medical institutions with limited testing conditions, and provides a universal decision-making tool for optimizing the postoperative management of children with BA.

At present, a multi-dimensional index framework has been established for the prediction system of NLS in children with BA, and many factors have been used to predict the survival of children with BA (4–10), including the age of KPE surgery, GGT, APRI, cytomegalovirus (CMV) infection, postoperative TBIL (13, 25, 26), JC, and postoperative cholangitis, which have been shown to be related to the long-term prognosis of children with BA after KPE.

Several studies have shown that rapid, early, and complete clearance of jaundice after Kasai surgery is the most valuable prognostic factor for long-term NLS (27, 28), and in addition, failure to achieve postoperative jaundice clearance has consistently been associated with shorter NLS and earlier liver transplantation in children (29). The results of multivariate analysis showed that failure to clear jaundice 3 months after surgery is a risk factor affecting 2-year NLS in children with BA (OR = 2.437), suggesting that successful bile drainage after surgery is the key to long-term native liver survival. This is consistent with the conclusion of the Ge (29) that jaundice that is not cleared after KPE is a risk factor for shorter NLS and earlier liver transplantation. Therefore, early postoperative monitoring of JC status and intervention are important strategies to improve prognosis.

Cholangitis is a common complication after KPE, with an incidence of 40%–90%, and its recurrence can further impair liver function by aggravating cholestasis and intrahepatic inflammation (30, 31). Poor clearance of jaundice indicates the possibility of cholestasis and hepatocellular damage, which may increase the risk of cholangitis (32). Each episode of cholangitis exacerbates liver fibrosis, worsens liver function, and induces various complications. Previous studies have shown a significant association between poor recovery of liver function and a higher incidence of cholangitis (33). Frequent episodes of cholangitis can further deteriorate liver function, creating a vicious circle (34) that ultimately leads to liver failure. Although no statistical association between cholangitis and NLS was found in this study, its pathophysiological role cannot be ignored, and the role and mechanism of cholangitis in NLS have been widely studied. They argue that cholangitis must be avoided as much as possible, especially in the early postoperative period, to maximize the success rate of PE and improve NLS (35, 36).

The age at surgery is also an important factor affecting NLS, and the later the operation age and the worse the postoperative liver function recovery, the shorter the NLS. The choice of age at surgery is inconclusive, but it is clear that KPE within 60 days of age has a positive effect on postoperative recovery and NLS (5). In this study, the rate of NLS was higher in children with ≤60 days of operation, and the results of multivariate analysis also showed that a later age at surgery is a risk factor affecting the 2-year NLS of children with BA (OR = 1.773), suggesting that early surgery could effectively block the process of biliary tract inflammation and delay the development of liver fibrosis. In addition, in this study, the liver fibrosis F4 stage was an independent risk factor for 2-year NLS. Only 12.5% of children with F4 stage achieved NLS, while the NLS rate of children with F1–F2 stage was significantly higher (75%), suggesting that the degree of liver fibrosis is the biological basis for determining the prognosis, and intervention in early liver fibrosis may improve the prognosis.

Through multivariate logistic regression analysis, this study confirmed that dynamic monitoring of GGT, ALB and APRI at 3 months after surgery had independent predictive value for the prognosis of children with BA, so we included them in the nomogram. GGT is mainly derived from hepatocytes and participates in the enterohepatic circulation of bile acids by catalyzing glutathione metabolism (37), and elevated serum GGT levels usually indicate cholestasis and a poor prognosis. Ahn et al. (38) reported that children with a serum GGT concentration of 550 IU/L at 5 months > months after KPE had a lower 5-year natural hepatic survival rate. In this study, the GGT at 3 months after surgery in the non-NLS group was significantly higher than that in the NLS group, suggesting that persistent cholestasis may accelerate liver injury. In this study, the level of ALB in the non-NLS group at 3 months after surgery was significantly lower than that in the NLS group, which was similar to the results of the multicenter study of Nightingale et al. (39): ALB in children with BA at 3 months after KPE<35 g/L is a poor prognostic indicator, which may be related to decreased hepatic synthetic function, imbalance of protein-energy metabolism, and increased systemic inflammatory consumption. APRI is a noninvasive indicator for assessing the degree of liver fibrosis in adults pioneered by Wai et al. (12), and has been used to monitor liver function in various chronic liver diseases. A number of scholars have studied the relationship between APRI and NLS time. Kim et al. (40) showed that children with higher APRI values had worse long-term survival outcomes. An analysis by Ahn et al. (38) concluded that APRI had a high predictive power of 5-year NLS rates in children with BA at four months after KPE. Multivariate logistic regression analysis in this study showed that a high APRI at 3 months postoperatively is an independent risk factor for 2-year native liver survival in children with BA (OR = 2.285), which further supported its potential as an early warning indicator in assessing prognosis.

However, the above parameters are usually analyzed individually. Therefore, a more accurate method combining these routine laboratory parameters is needed to predict the prognosis of infants with BA. So far, there are few prognostic prediction models for children with BA within 2 years based on indicators within 3 months after KPE. We combine liver histopathological features with dynamic biochemical marker monitoring, and all predictors can be conveniently obtained from regular examinations while ensuring the predictive validity of the model (liver biopsy is performed during the KPE). The nomogram constructed in the study can be used to calculate the score for each independent predictor, and the sum of the scores corresponds to the predicted probability of a poor prognosis. Our study was validated internally, and the nomogram had good identification and calibration capabilities, which significantly enhanced its feasibility for routine clinical implementation. The prediction model can provide a quantitative decision-making basis for the adjustment of postoperative management strategies and the timing of liver transplantation.

Existing prognostic models have many limitations: some scholars have established nomograms based on gallbladder morphology and serum TBIL and total protein levels, but the diagnostic value is moderate (AUC = 0.673) (41). However, although the model combining genetic features and elastography has high accuracy (42), it is difficult to promote in primary hospitals due to the cost of testing and the availability of equipment. Some scholars have used liver stiffness measurement (LSM) to assess prognosis (11) and found that LSM at three months was not satisfactory in predicting the prognosis within two years of KPE. This may be due to the fact that LSM is also affected by factors such as inflammation, hepatocyte swelling, and tissue edema (43), and the predictive performance is unstable. These models are limited by medical resources and cannot be widely used in primary hospitals.

In contrast, this study innovatively incorporated dynamic biochemical indicators into the prediction model at 3 months after surgery, which offers several notable advantages. First of all, all indicators are from routine laboratory tests, without special equipment or complex operations, which significantly lowers the threshold for clinical application. Secondly, 3 months after surgery is the critical window period for liver function compensation, and abnormal indicators at this time can indicate the progression of fibrosis in the early stage, providing a time window for intervention. Finally, DCA showed that the clinical net benefit of nomogram was significantly better than that of “full intervention” or “no intervention” strategy within the threshold probability range of 10%–50%, providing a quantitative basis for individualized treatment decisions. In addition, internal validation confirms that the model has good calibration capabilities, further enhancing its applicability in resource-limited areas.

In addition, this study further confirmed the robustness and generalizability of the nomogram model through age subgroup analysis and training-test set validation. Within the age subgroups, the model demonstrated stable predictive performance in children with surgical age ≤60 days and >60 days (AUCs of 0.926 and 0.867, respectively). In the training-test set validation, the model performed excellently in both the training set (AUC = 0.872) and the test set (AUC = 0.971), with good calibration and no obvious overfitting. Most predictive factors showed consistent directions and significance of OR values across the two sets, further supporting the reliability of the model structure. Although “jaundice clearance failure” did not reach statistical significance in the ≤60 days subgroup (*P* = 0.097) or in the test set (*P* = 0.053), its relatively high OR still holds clinical relevance. In summary, the age subgroup analysis and training-test set validation together indicate that this nomogram model is a robust, reliable prognostic assessment tool suitable for different clinical scenarios.

This study has the following limitations: First, as a single-center retrospective study, the model was only internally validated using the Bootstrap method, lacking validation in multi-center external cohorts, so its generalizability needs further confirmation. Second, due to the absence of precise event timing in data collection, we used binary logistic regression rather than survival analysis, which limits the exploration of dynamic effects of factors. In addition, the model did not include dynamic clinical events such as cholangitis episodes, which may affect the comprehensiveness of the predictions. Future work requires prospective, multi-center studies that collect time-to-event data and incorporate more clinical variables to enhance the clinical applicability of the model.

## 5 Conclusions

In conclusion, this study constructed and validated a non-invasive nomogram based on liver fibrosis stage, surgical age, jaundice clearance and postoperative dynamic biochemical indicators, and its high predictive performance (AUC = 0.872) and clinical applicability provided an important tool for the individualized management of children with BA. This model can provide a quantitative decision-making basis for the adjustment of postoperative management strategies and the timing of liver transplantation. Future studies should focus on optimizing the model architecture through multidisciplinary collaboration and validating its generalizability in multicenter cohorts.

## Data Availability

The raw data supporting the conclusions of this article will be made available by the authors, without undue reservation.
